# Canine Fibroblast Growth Factor 21 Ameliorates Hyperglycemia Associated with Inhibiting Hepatic Gluconeogenesis and Improving Pancreatic Beta-Cell Survival in Diabetic Mice and Dogs

**DOI:** 10.1371/journal.pone.0155598

**Published:** 2016-05-20

**Authors:** Pengfei Xu, Yingjie Zhang, Xinghao Jiang, Junyan Li, Liying Song, Mir Hasson Khoso, Yunye Liu, Qiang Wu, Guiping Ren, Deshan Li

**Affiliations:** Biopharmaceutical Lab, School of Life Science, Northeast Agricultural University, Harbin, P.R. China; The University of Melbourne, AUSTRALIA

## Abstract

Diabetes mellitus is a common endocrinopathy in dog. Fibroblast growth factor 21 (FGF-21) is a secreted protein, which is involved in glucose homeostasis. We speculate that the recombinant canine FGF-21 (cFGF-21) has the potential to become a powerful therapeutics to treat canine diabetes. The cFGF-21 gene was cloned and expressed in *E*. *coli* Rosetta (DE3). After purification, a cFGF-21 protein with the purity exceeding 95% was obtained. Mouse 3T3-L1 adipocytes and type 1 diabetic mice/dogs induced by STZ were used to examine the biological activity of cFGF-21 *in vitro* and *in vivo*, respectively. Results showed that cFGF-21 stimulated glucose uptake in adipocytes significantly in a dose-dependent manner, and reduced plasma glucose significantly in diabetic mice/dogs. After treatment with cFGF-21, the serum insulin level, glycosylated hemoglobin (HbA1c) level and the expressions of the hepatic gluconeogenesis genes (glucose-6-phosphatase, G6Pase and phosphoenolpyruvate carboxykinase, PCK) of the diabetic mice/dogs were attenuated significantly. In the mouse experiment, we also found that the phosphorylation of signal transducer and activator of transcription 3 (STAT3) and the expression of suppressor of cytokine signaling 3 (SOCS3) were up-regulated significantly in the livers after treatment. Histopathological and immunohistochemical results showed that treatment with cFGF-21 promoted recovery of pancreatic islets from STZ-induced apoptosis. Besides, we also found that treatment with cFGF-21 protected liver against STZ or hyperglycemia induced damage and the mechanism of this action associated with inhibiting oxidative stress. In conclusion, cFGF-21 represents a promising candidate for canine diabetes therapeutics. The mechanism of cFGF-21 ameliorates hyperglycemia associated with inhibiting hepatic gluconeogenesis by regulation of STAT3 signal pathway and improving pancreatic beta-cell survival.

## Introduction

Diabetes mellitus (DM) is an endocrine disease of the dog characterised by hyperglycaemia, glycosuria and weight loss, which is caused by an absolute or relative deficiency in the pancreatic beta-cell hormone insulin [1]. The pathogenesis of DM in dogs is like the pathogenesis of human type 1 diabetes, but the onset period is unlike human type 1 diabetes, which occurs primarily in adolescence and early adulthood, while most of canine DM occurs in middle-aged and older dogs [1–3]. The incidence of diabetes mellitus has been steadily rising in dogs [4,5]. Nowdays, the drug commonly used in canine diabetes mellitus is human used insulin. Just like in the UK, the only insulin product consisting of purified porcine insulin is available for treatment of DM in dogs [6]. Since the supply of bovine and porcine insulin for veterinary use generally relies on human market availability, insulin for diabetic dogs is likely to become limited [6]. Besides, insulin for treating canine diabetes exhibits some disadvantages, such as causing hypoglycemia and daily injection. Therefore, developing a new drug with long-acting hypoglycemic effect which designed for dogs specifically is necessary.

FGF-21, which was first reported by Nishimura et al. in 2000, is an atypical member of the FGF super family, which is specifically expressed in the liver and functions as an endocrine hormone [7–9]. When the recombinant FGF-21 protein is injected to ob/ob, db/db, and diet-induced obese (DIO) mice, which are models of insulin resistant, it reduces blood glucose and triglycerides to near normal levels and ameliorates insulin resistance [10–13]. Besides, previous study has demonstrated that recombinant human and mouse FGF-21 can ameliorate type 1 diabetes in mouse model [14]. Therefore, the hypothesis was that the cFGF-21 protein had the ability to ameliorate the diabetes mellitus in dogs. The objective of the present study was to develop cFGF-21, whereby using gene engineering method, to express this recombinant cFGF-21 protein in bacteria and to assess its activity via stimulating glucose uptake in mouse 3T3-L1 adipocytes and type 1 diabetic mice/dogs.

## Materials and Methods

### Construction of pSUMO-cFGF21

Canine FGF-21 sequence (GenBank: XM_014116979.1) which contained restriction enzyme sites of Bsa I and Not I was synthesized by Invitrogen. pSUMO vector (LifeSensors Inc.) whose multiple cloning site contained restriction enzyme sites of Bsa I and Not I was used to express the cFGF-21 protein. The pSUMO vector and the synthesized cFGF-21 sequence were digested via restriction enzyme sites of Bsa I and Not I, then ligating the pSUMO vector and cFGF-21 sequence via restriction enzyme sites. Finally, a recombinant plasmid pSUMO-cFGF21 was obtained. Authenticity of the inserted fragment was confirmed by restriction enzyme analysis and automated DNA sequencing.

### Expression and Purification of cFGF-21

The recombinant plasmid, pSUMO-cFGF21 was transformed into host *E*. *coli* Rossetta (DE3). A single colony was grown in LB media containing ampicillin (100 μg/mL). When OD_600_ reached 0.4 to 0.6, IPTG was added into the medium to the final concentration of 0.25 mmol/L, and the *E*. *coli* continued growing at 25°C for 10 h to induce the expression of cFGF-21 protein. The cFGF-21 was purified by a Ni Sepharose 6 Fast Flow column in AKTA Purifier (GE Healthcare). Finally, cFGF-21 was stored in PBS buffer. The endotoxin was removed from the purified cFGF-21 by ToxinEraserTM Endotoxin Removal Kit (GenScript), and the purity of cFGF-21 was analyzed by SDS-PAGE and high performance liquid chromatography (HPLC).

### Glucose Uptake Activity Assay of cFGF-21

Differentiated mouse 3T3-L1 adipocytes (Chinese Cell Center, China) as an adipocyte model to detect the glucose regulation activity of cFGF-21. 3T3-L1 adipocytes were grown in Dulbecco’s Modified Eagle’s Medium (DMEM; GIBCO/BRL, Gaithersburg, MD), supplemented with 10% new-born calf serum (GIBCO/BRL) containing penicillin, streptomycin, and gentamycin in the presence of 5% CO_2_ at 37°C. The cells were starved for 12 h in a serum-free medium followed by stimulation without or with various concentrations of cFGF-21 (10, 100, and 1000 nmol/L) for another 24 h. The glucose consumption of the medium was examined by mini-glucose oxidase-peroxydase (GOD-POD) assay kit (Beijing Kingkawk Pharmaceutical CO., LTD) according to the manufacturer's protocol. Absorbance at 490 nm was recorded, and the glucose consumption rate was calculated.

### Animals

All procedures involving mice and dogs were carried out with prior approval from the Animal Care and Use Committee of Institute of Materia Medica, China. Male C57BL/6 mice (SPF) weighing 25 to 30 g were purchased from the Experimental Animal Center of ChangChun YiSi Company. The mice were acclimated and housed individually in standard-sized cages with enough nesting material—sawdust in a temperature and humidity-controlled (Temperature: about 23°C; relative humidity: about 50%), pathogen-free room on a 12 h light cycle with free access to food and water. The mice were used for induction of type 1 diabetes with injecting streptozotocin (STZ, Sigma Chemical Co) which was dissolved sterile citrate buffer (0.05 mol/L sodium citrate, pH 4.5, 45 mg/kg) intraperitoneally at dose of 50 mg/kg, and citrate buffer was injected into their male littermates (control). STZ or citrate buffer was injected into the mice for 5 consecutive days. One week after the fifth injection, the blood glucose of the mice was measured for 3 consecutive days, and the mice with blood glucose level above 16.65 mmol/l were deemed to be diabetic [15]. Male dogs (beagles, approximately 2 years old) were purchased from Institute of Shenyang Kangping Laboratory Animal. Dogs were housed in cages (Length/Width/Height: 100cm/100cm/100cm, each cage accommodated one dog) in a temperature and humidity-controlled (Temperature: about 23°C; relative humidity: about 50%), pathogen-free room on a 12 h light cycle with free access to water, toy ball and toy bone, but access to food at special time. All of these dogs were taken out for a walk for 1 hour in one enclosed courtyard. The health condition of these animals were observed by one veterinarian once a week. Diabetes dogs were induced by STZ at a dose of 25 mg/kg (10 mmol/L Na-citrate buffer, pH 4.5) [16]. STZ was injected into the dogs subcutaneously for 2 times, and the second injection of STZ was inject into the dogs 2 weeks after the first injection. The fasting blood glucose (FBG) of the dogs exceeded 11.1 mmol/L continuously and the serum insulin leves decreased significantly were thought as diabetic dogs. In the mouse experiment, mice were divided into three groups (each group contained 8 mice), i.e. (1) healthy mice treated with PBS (normal control), (2) diabetic mice treated with PBS (model control) and (3) diabetic mice treated with cFGF-21 at a dose of 1.0 mg/kg (cFGF-21 group). cFGF-21 was injected into the diabetic mice subcutaneously once daily for one month. The blood glucose of the mice in different group was measured every three days. An oral glucose tolerance test (OGTT) was performed at the end of the treatment period. The mice with free access to water were fasted for 6 hours, then given glucose by gavage at dose of 2 g/kg. The blood glucose was measured at 0, 30, 60, 90 and 120 min post gavage. The mice were euthanasia using pentobarbital sodium at the end of the experiment. Blood samples were taken from each mouse of three groups for HbA1c and serum insulin measurements (using mouse insulin ELISA kit and HbA1c ELISA kit which were purchased from R&D, USA). The liver and the pancreas of each mouse of three groups were removed for qPCR, western blotting, H&E staining and immunohistochemical staining. In the dog experiment, dogs were divided into three groups (each group contained 3 dogs), i.e. (1) healthy dogs treated with PBS (normal control), (2) diabetic dogs treated with PBS (model control) and (3) diabetic dogs treated with cFGF-21 at a dose of 0.5 mg/kg (cFGF-21 group). cFGF-21 was injected into the diabetic dogs subcutaneously once daily for 12 days. The FBG of the dogs in different group was measured every three days. Then the dogs were euthanasia using pentobarbital sodium. Blood samples were taken from each dog of three groups for serum insulin measurement, and the liver of each dog of three groups were removed for qPCR. No experimental mouse and dog died unexpectedly prior to the experimental endpoint.

### H&E Staining

The livers and pancreases of the experimental animals were removed. One lobe of the pancreas was preserved in buffered formaldehyde and embedded in paraffin. 5 μm sections were cut and stained with hematoxylin and eosin. The other livers were flash frozen in liquid nitrogen and stored at −80°C for RNA analysis.

### Immunohistochemistry Analysis

Pancreases were fixed in 4% paraformaldehyde solution, embedded in paraffin, and sectioned at 5 μm. After dehydration, sections were subjected to antigen retrieval in 0.01 mol/L citrate buffer (pH 6.0) by microwaving, and then placed in 3% hydrogen peroxide in methanol for 30 min at room temperature. After blocking with 5% BSA, the sections were incubated with anti-insulin antibody (1:200, Cell Signaling Technology Inc., Danvers, MA) overnight at 4°C, followed by the appropriate secondary antibody (1:200, Cell Signaling Technology Inc., Danvers, MA). The reaction was visualized with DAB solution. After counterstaining with hematoxylin, the sections were dehydrated and viewed under a light microscope.

### Measurement of Reactive Oxygen Species (ROS), Malondialdehyde (MDA), and Antioxidant Enzymes in Livers and Pancreases

Liver and pancreatic tissues were lysed for total protein extraction in radioimmunoprecipitation assay (RIPA) buffer (Nanjing Jiancheng Bioengineering Institute, China) together with a protease inhibitor PMSF (Sigma-Aldrich Corporation, Saint Louis, MO, USA). ROS and MDA contents were measured by ROS Assay Kit and MDA assay kit which were purchased from Nanjing Jiancheng Bioengineering Institute, China. The activities of antioxidant enzymes, namely total superoxide dismutase (T-SOD), catalase (CAT), glutathione reductase (GR) and Glutathione peroxidase (GSH-Px), were measured by T-SOD assay kit, CAT assay kit, GR assay kit and GSH-Px assay kit which were purchased from Nanjing Jiancheng Bioengineering Institute, China.

### Serum Alanine Aminotransferase (ALT), Aspartate Aminotransferase (AST) and Alkaline Phosphatase (ALP)

Serum of the mice in each group was collected at the end of the experiment, and ALT, AST, ALP and GGT were evaluated in samples of serum. The activity was evaluated in Harbin Electricity Hospital, Harbin, China.

### Reverse Transcription and Real-Time PCR

Total RNA was isolated from cells and tissues (50–100 mg) using TRIZOL (Invitrogen, Carlsbad, CA) according to the manufacturer's instructions. Reverse transcription and quantitative PCR were performed using M-MLV Platinum RT-qPCR Kit (Invitrogen, Carlsbad, CA). Realtime was carried out using the Eppendorf Realplex 4 instrument (Eppendorf, Hamburg, Germany). Primers for genes were synthesized in Invitrogen (Invitrogen, Shanghai, China) and the primer sequences used were shown as Table 1.

**Table 1:** Sequences of PCR primers used in this study.

	Gene	Primer Sequence
Mouse	G6Pase	F 5'-TCAACCTCGTCTTCAAGTGGATT-3'
		R 5'-GCTGTAGTAGTCGGTGTCCAGGA-3'
	PCK	F 5'-GGCGGAGCATATGCTGATCC-3'
		R 5'-CCACAGGCACTAGGGAAGGC-3'
	STAT3	F 5'-CTTGGCCCTTTGGAATGA-3'
		R 5'-TCTCGCTGAAGCGCAGTA-3'
	SOCS3	F 5'-GGGTGGCAAAGAAAAGGAG-3'
		R 5'-GTTGAGCGTCAAGACCCAGT-3'
	CAT	F 5'-AATCCTACACCATGTCGGACA-3'
		R 5'-CGGTCTTGTAATGGAACTTGC-3'
	Sod2	F 5'-TCCCAGACCTGCCTTACGATAT-3'
		R 5'-GGTGGCGTTGAGATTGTTCA-3'
	GCL-c	F 5'-GTTATGGCTTTGAGTGCTGCAT-3'
		R 5'-ATCACTCCCCAGCGACAATC-3'
	Gpx-1	F 5'-CCAGGAGAATGGCAAGAATGA-3'
		R 5'-TCTCACCATTCACTTCGCACTT-3'
	Bcl2	F 5'-GGTGGTGGAGGAACTCTTCA-3'
		R 5'-ATGCCGGTTCAGGTACTCAG-3'
	Bax	F 5'-TGCAGAGGATGATTGCTGAC-3'
		R 5'-GATCAGCTCGGGCACTTTAG-3'
	beta-actin	F 5'- ACATCTGCTGGAAGGTGGAC-3'
		R 5'-GGTACCACCATGTACCCAGG-3'
Dog	G6Pase	F 5'-TGAAACTTTCAGCCACATCCG-3'
		R 5'-GCAGGTAAAATCCAAGTGCGAA-3'
	PCK	F 5'-AGCTTTCAATGCCCGATTTCCAGG-3'
		R 5'-TCAGCTCGATGCCGATCTTTGACA-3'
	GAPDH	F 5'-ACAGTCAAGGCTGAGAACGG-3'
		R 5'-CCACAACATACTCAGCACCAGC-3'

### Western Blotting

Tissues or cells were lysed for total protein extraction in radioimmunoprecipitation assay (RIPA) buffer (Nanjing Jiancheng Bioengineering Institute, China) together with a protease inhibitor PMSF (Sigma-Aldrich Corporation, Saint Louis, MO, USA). Proteins were separated by SDS-poly-acrylamide gel electrophoresis and transferred to polyvinylidene difluoride membranes (Amersham Life Science, Piscataway, NJ). Membranes were incubated for 2 h in PBS blocking buffer, and then probed with antibodies. Anti-STAT3, anti-pSTAT3 (Tyr705), anti-SOCS3 and anti-Nrf2 antibodies were purchased from Cell Signaling Technology Inc., Danvers, MA; anti-G6Pase antibody was purchase from Santa Cruz; anti-PCK, anti-Bcl2 and anti-Bax antibodies were purchased from Sangon Biotech Co., Ltd, China. The membranes were washed three times for 10 min in PBST then incubated in secondary antibody diluted in TBS blocking buffer for 1 h at room temperature. Membranes were developed using the ECL reagents, Amersham (Pittsburgh, PA). Blots were developed using an ECL kit (Amersham Biosciences, Piscataway, NJ) and exposed with Blue Basic Autorad Film (ISC BioExpress).

### Statistical Analysis

All data were performed using one-way analysis of variance (ANOVA), followed by the Student two-tailed t test. Statistical significance was defined as P < 0.05.

## Results

### Preparation of cFGF21

**Fig 1:**
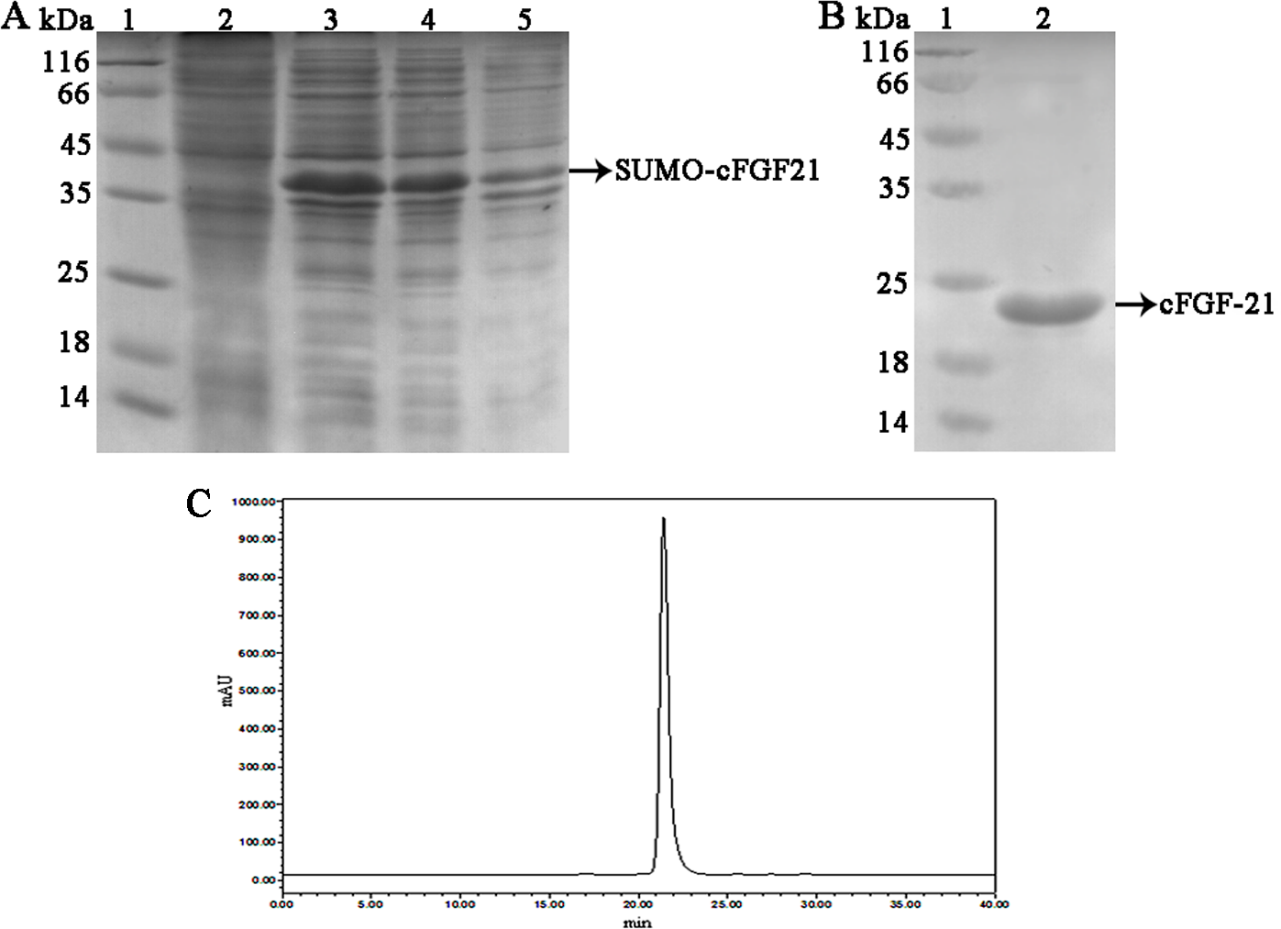
Preparation of cFGF-21. (A) SDS-PAGE gel analysis of cFGF-21 expression. Lane 1: Protein molecular mass marker; lane 2: The lysates of Rosetta (DE3) cells containing pSUMO-cFGF21 without induced by IPTG; Lane 3: The lysates of Rosetta (DE3) cells containing pSUMO-cFGF21 with induced by IPTG; Lane 4: The supernatant of Rosetta (DE3) cells containing pSUMO-cFGF21 with induced by IPTG; Lane 5: The precipitation of Rosetta (DE3) cells containing pSUMO-cFGF21 with induced by IPTG. (B) SDS-PAGE gel analysis of purified PEG-FGF21. The cFGF21 protein was purified by a Ni Sepharose 6 Fast Flow column. Lane 1: Protein molecular mass marker; lane 2: cFGF-21. (C) The purity of cFGF-21. The purity of cFGF-21 was analyzed by HPLC.

The construction of recombinant plasmid pSUMO-cFGF21 used gene engineering method. Authenticity of cFGF-21 sequence was confirmed by restriction enzyme analysis and automated DNA sequencing. Results showed that an authentic recombinant plasmid pSUMO-cFGF21 was obtained (data not shown). The pSUMO-cFGF21 plasmid was transformed into *E*. *coli* Rossetta (DE3) to express the protein, and soluble proteins exceeded 70% of all (Fig 1A). The protein was purified by affinity chromatograph of Ni^2+^, and the purity of cFGF-21 was evaluated by HPLC. Finally, cFGF-21 protein with purity exceeding 95% was obtained (Fig 1B and 1C).

### cFGF-21 Exhibits Glucose Uptake Activity in 3T3-L1 Cells and Hypoglycemic Effect in Diabetic Mice

**Fig 2:**
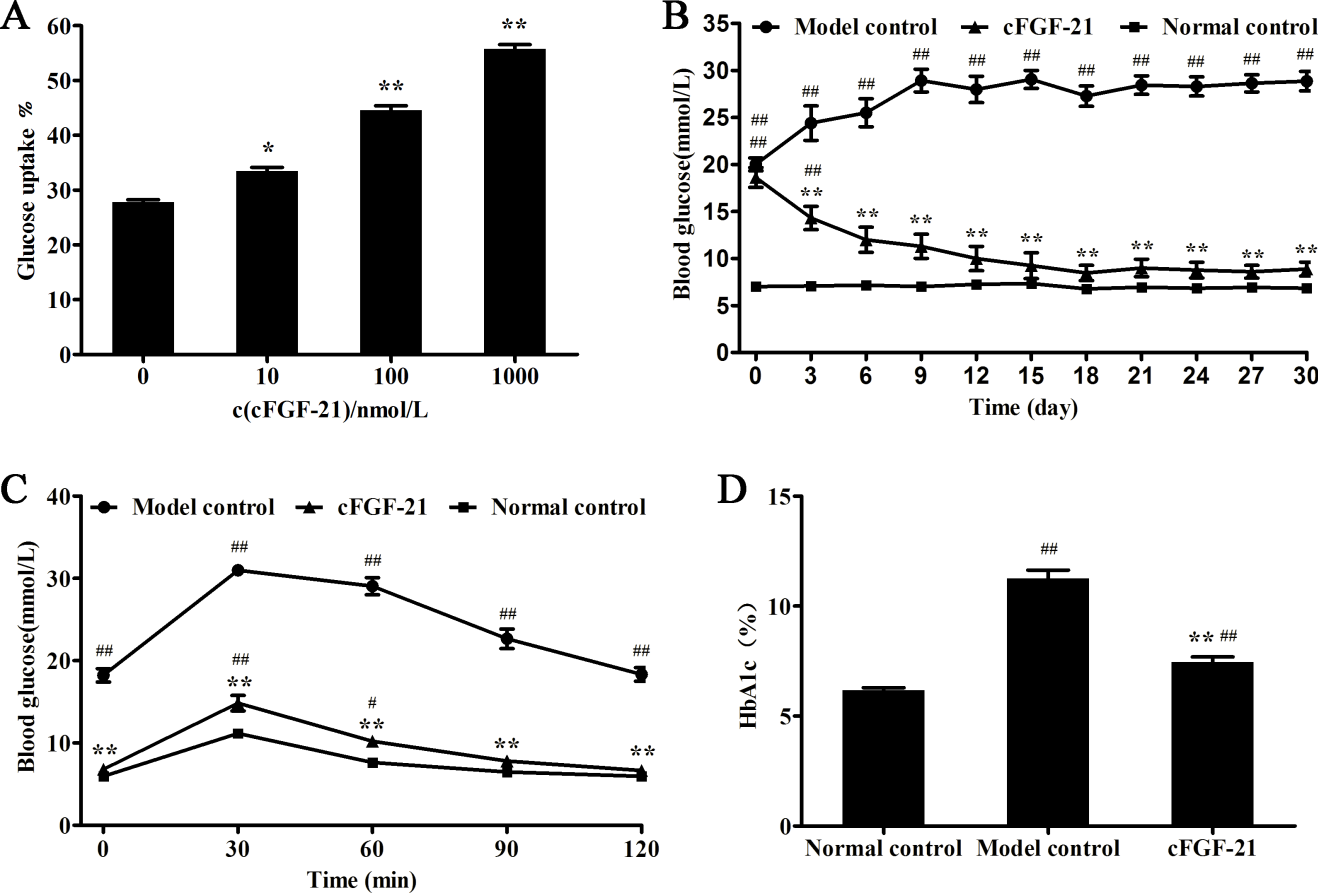
cFGF-21 exhibits good activity *in vitro* and *in vivo*. (A) cFGF-21 improves the glucose uptake of 3T3-L1. 3T3-L1 cells were starved for 12 h in a serum-free medium followed by stimulation without or with various concentrations of cFGF-21 for another 24 h. The glucose consumption of the medium was examined by GOD-POD assay kit, *p<0.05, **p<0.01. (B) cFGF-21 reduces blood glucose of the mice significantly. Blood glucose levels of mice in each group were monitored once every three days at 8:00 a.m. before treatment. (C) Glucose profiles of OGTT. (D) The HbA1C level. Data represent the mean±SD. significant as compared to model control, *p<0.05, **p<0.01; significant as compared to normal control, ^#^p<0.01, ^##^p<0.01.

After 3T3-L1 adipocytes treated with cFGF-21, the glucose uptake of the cells was improved significantly in a dose-dependent manner (Fig 2A). Blood glucose levels of mice in each group were monitored once every three days at 8:00 a.m. before treatment. As shown in Fig 2B, compared to model group, cFGF-21 reduced blood glucose of diabetic mice significantly and exhibited good long-acting hypoglycemic effects. The result of OGTT showed that treatment with cFGF-21 improved glucose tolerance significantly compared with model control (Fig 2C). Besides, treatment with cFGF-21 reduced the HbA1c level significantly compared with model control (Fig 2D).

### cFGF-21 Inhibits Hepatic Gluconeogenesis in the Diabetic Mice

**Fig 3:**
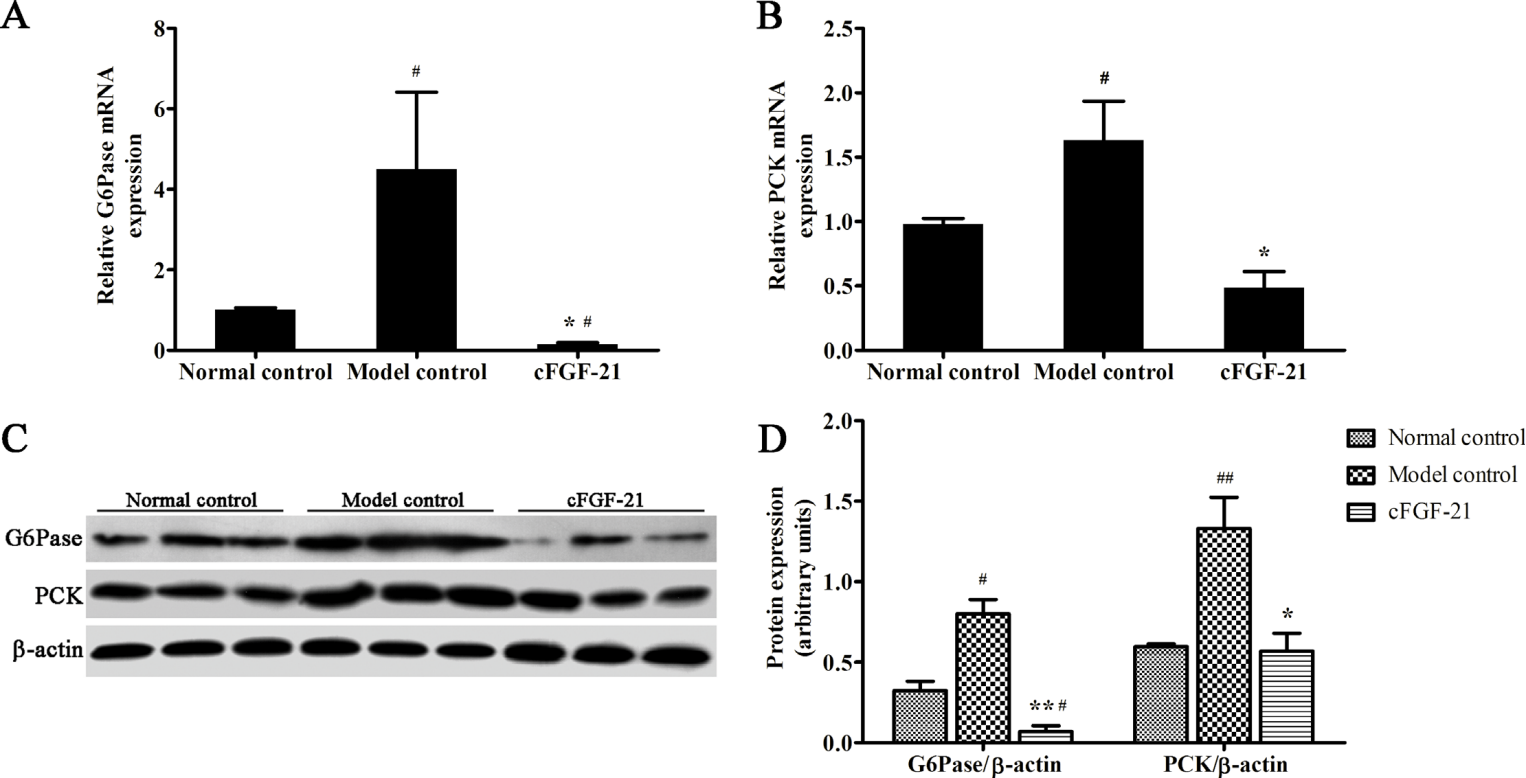
cFGF-21 inhibits the expressions of G6Pase and PCK. (A) Realtime PCR analysis of G6Pase. (B) Realtime PCR analysis of PCK. (C) Western blot analysis of G6Pase and PCK. (D) The relative G6Pase and PCK levels. The relative G6Pase and PCK levels were expressed as the ratio G6Pase/beta-actin and PCK/beta-actin. The bands were derived from three blots and analyzed with Image pro plus. The data were performed using one-way analysis of variance (ANOVA), followed by Student two-tailed t test. Data represent the mean±SD. significant as compared to model control, *p<0.05, **p<0.01; significant as compared to normal control, ^#^p<0.01, ^##^p<0.01.

To assess the effects of cFGF-21 on hepatic gluconeogenesis, qPCR and western blotting were used to analyze expression of the two key enzymes on hepatic gluconeogenesis, namely, G6Pase and PCK in livers of the mice. Results showed that the expressions of G6Pase and PCK in model control were up-regulated significantly compared with normal control. However, treatment with cFGF-21 down-regulated the expressions of G6Pase and PCK significantly compared with model control. Besides, the expression of G6Pase in cFGF-21 treatment group was lower than normal control (Fig 3).

### cFGF-21 Inhibits Hepatic Gluconeogenesis Associated with STAT3 Signal Pathway in Diabetic Mice

**Fig 4:**
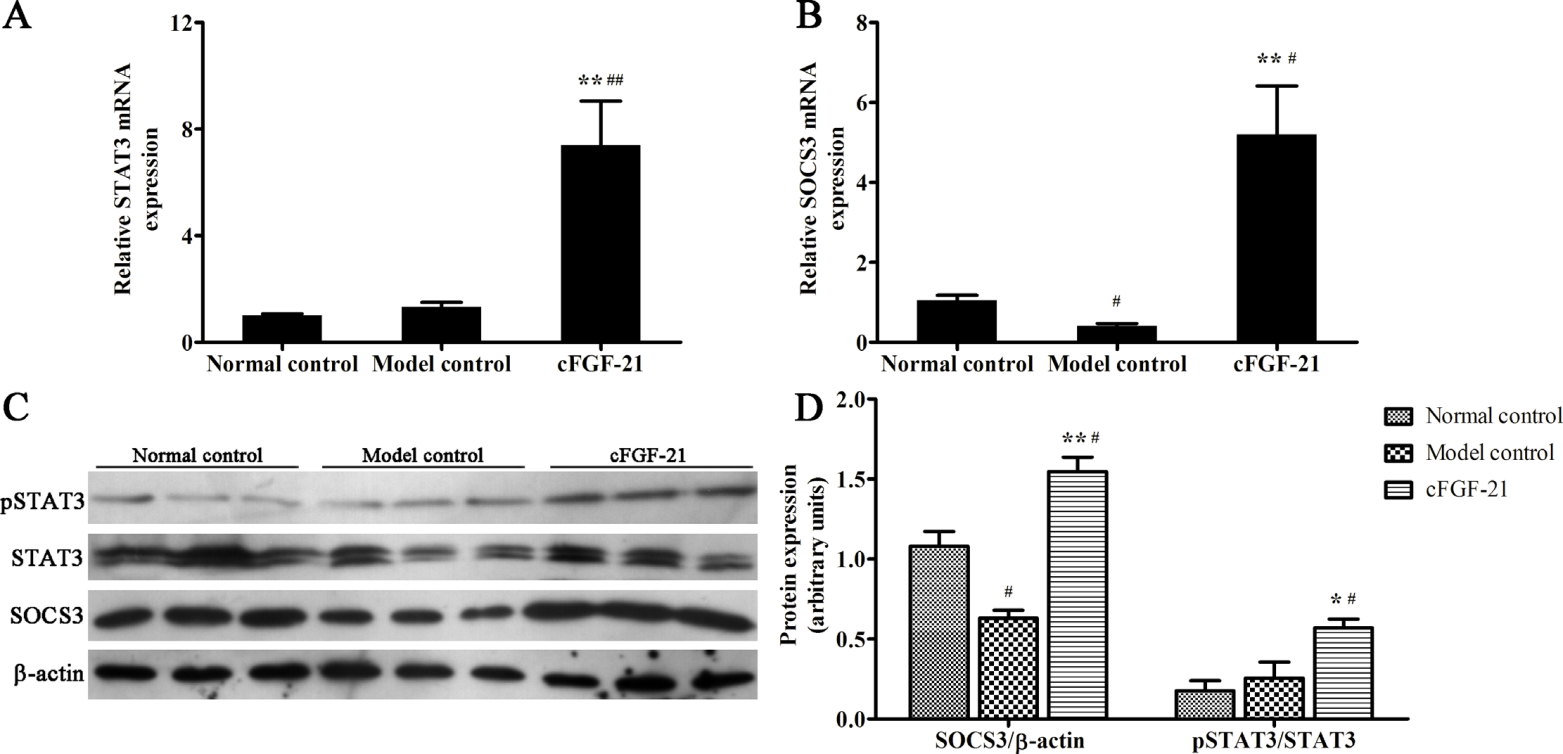
cFGF-21 improves the phosphorylation of STAT3 and the expression of SOCS3. (A) Realtime PCR analysis of the expression of STAT3. (B) Realtime PCR analysis of the expression of SOCS3. (C) Western blot analysis of the phosphorylation of STAT3 and the expression of SOCS3. (D) The relative the phosphorylation of STAT3 and the expression of SOCS3 levels. The relative the phosphorylation of STAT3 and the expression of SOCS3 levels were expressed as the ratio pSTAT3/STAT3 and SOCS3/beta-actin. The bands were derived from three blots and analyzed with Image pro plus. The data were performed using one-way analysis of variance (ANOVA), followed by Student two-tailed t test. Data represent the mean±SD. significant as compared to model control, *p<0.05, **p<0.01; significant as compared to normal control, ^#^p<0.01, ^##^p<0.01.

To investigate the mechanism of cFGF-21 inhibiting hepatic gluconeogenesis. qPCR and western blotting were used to measure the expressions of STAT3, SOCS3 and the phosphorylation of STAT3 in the livers of the mice. qPCR results showed that the expression of SOCS3 in model control decreased significantly compared with normal control, and there were no significant difference of the STAT3 expression between normal control and model control, whereas cFGF-21 up-regulated the expressions of STAT3 and SOCS3 signifiantly compared with model control (Fig 4A and 4B). Western blotting showed that the expression of SOCS3 in model control decreased significantly compared with normal control, and there was no significant difference phosphorylation of STAT3 between normal control and model control, whereas treatment with cFGF-21 enhanced the expression of SOCS3 and the phosphorylation of STAT3 significantly compared with model control (Fig 4C and 4D).

### cFGF-21 Attenuates STZ Induced Pancreatic Damage in Diabetic Mice

**Fig 5:**
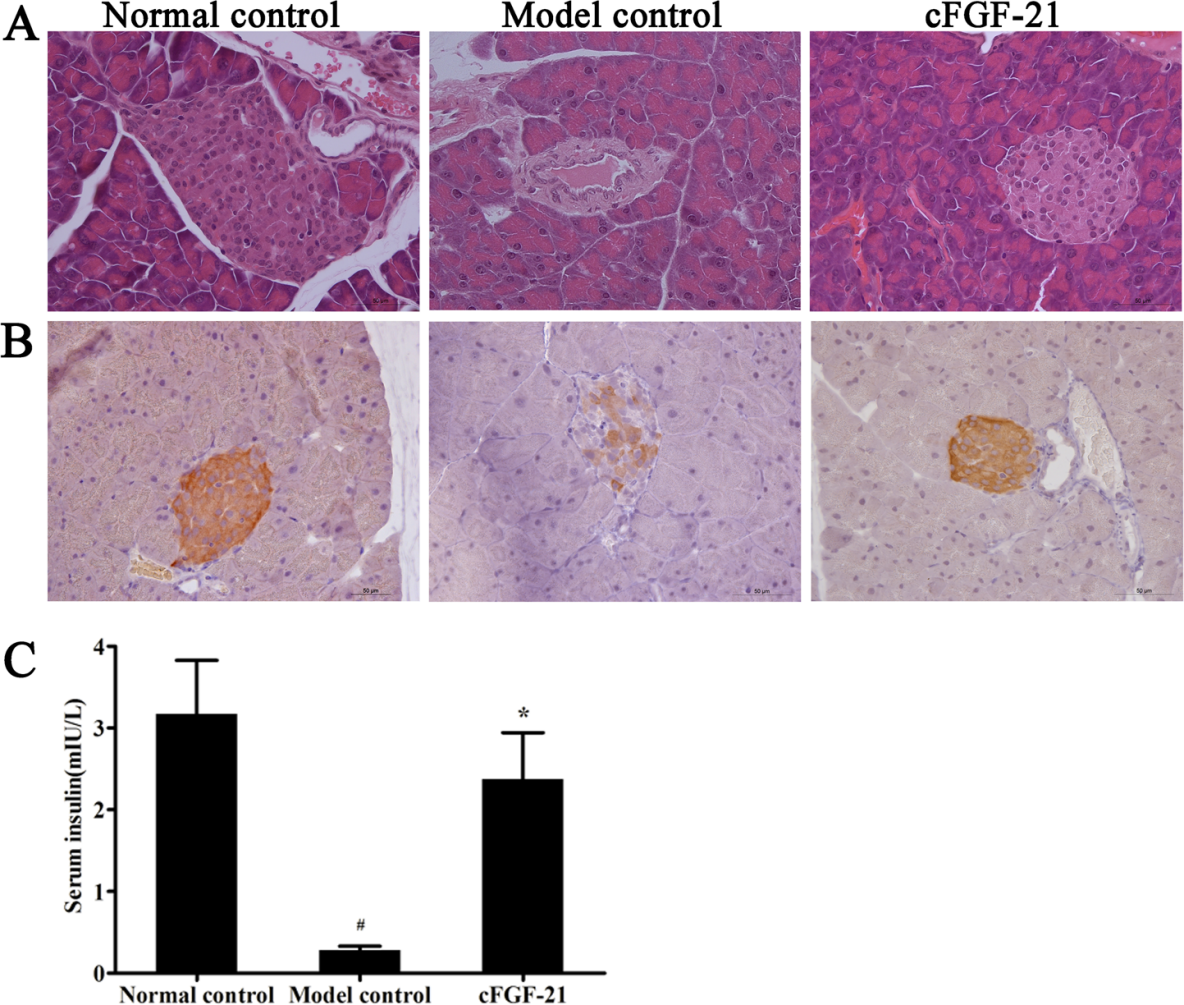
cFGF-21 attenuates STZ induced pancreatic damage. (A) H&E staining of pancreas. (B) Immunohistochemistry analysis of pancreas. Representative histology (40X magnification; bar = 50 mm) and correlation between bioluminescent signal and histological measure of beta-cell mass from the mice in normal control, model control and cFGF-21 group. (C) The insulin level of the mice in different groups. Blood samples were taken from each mouse of three groups when the mice sacrificed, and the serum insulin level was measured by mouse insulin ELISA kit. Data represent the mean±SD. significant as compared to model control, *p<0.05; significant as compared to normal control, #p<0.01.

To evaluate the effect of cFGF-21 on STZ induced pancreatic damage, H&E staining and immunohistochemistry for the pancreases of the mice were done; the serum insulin level of the mice was measured. Results of H&E staining and immunohistochemistry showed that exposure to STZ caused disruption of the pancreatic islets of Langerhans, but treatment with cFGF-21 restored this change gradually and saved islet cells (beta-cells) from damage (Fig 5A and 5B). Besides, the insulin level in the cFGF-21 group was higher than the model group, of which insulin level decreased significantly compared with normal control (Fig 5C).

### cFGF-21 Inhibits STZ Induced Apoptosis and Oxidative Stress in the Pancreas in Diabetic Mice

**Fig 6:**
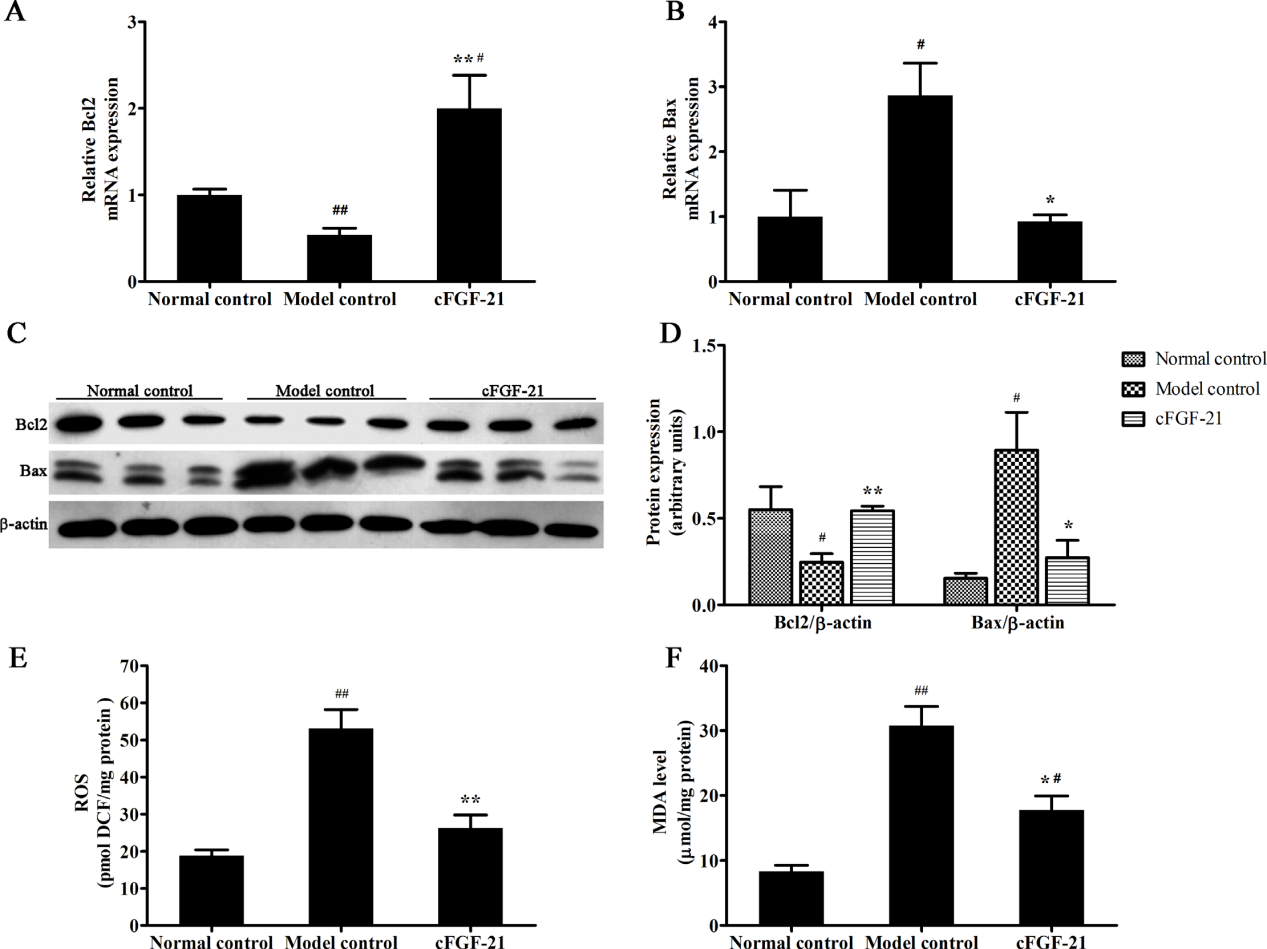
cFGF-21 attenuates the expressions of Bcl2 and Bax and inhibits STZ-induced oxidative stress in pancreases. (A) Realtime PCR analysis of the expression of Bcl2. (B) Realtime PCR analysis of the expression of Bax. (C) Western blot analysis of the the expressions of Bcl2 and Bax. (D) The relative expressions of Bcl2 and Bax levels. The relative the expressions of Bcl2 and Bax levels were expressed as the ratio Bcl2/beta-actin and Bax/beta-actin. The bands were derived from three blots and analyzed with Image pro plus. (E) The ROS content in pancreases. (F) The MDA content in pancreases. The data were performed using one-way analysis of variance (ANOVA), followed by Student two-tailed t test. Data represent the mean±SD. significant as compared to model control, *p<0.05, **p<0.01; significant as compared to normal control, ^#^p<0.01, ^##^p<0.01.

To assess the effect of cFGF-21 on STZ-induced apoptosis in pancreases, the expression of apoptosis-related genes (Bcl2 and Bax) were measured via qPCR and western blotting. Results showed that the expression of Bcl2 in model control decreased significantly compared with normal control; the expression of Bcl2 in cFGF-21 group was up-regulated significantly compared with model control; the expression of Bax in model control increased significantly compared with normal control and the expression of Bax in cFGF-21 group decreased significantly compared with model control (Fig 6A–6D). To assess the effect of cFGF-21 on STZ-induced oxidative stress in pancreases, the ROS/MDA content in pancreases and the activities of some antioxidative enzymes were measured. Results showed that the ROS and MDA contents in model control increased significantly compared with normal control. After treatment with cFGF-21, the ROS and MDA contents in pancreases decreased significantly compared with model control (Fig 6E and 6F). Besides, the activities of the antioxidative enzymes decreased significantly in model control compared with normal control, whereas the activities of the antioxidative enzymes increased significantly in cFGF-21 group compared with model control (Table 2).

**Table 2:** Impact of cFGF-21 on pancreatic activities of antioxidative enzymes in mice.

	SOD	CAT	GR	GSH-Px (U/mg protein)
Normal control	104.52±6.27	41.37±3.06	292.42±10.76	624.84±31.72
Model control	43.03±3.59^##^	20.61±2.79^##^	167.55±7.50^##^	251.07±16.77^##^
cFGF-21	117.10±6.94**	32.57±2.25*^#^	252.88±17.37**^#^	489.02±24.43**^#^

The values (x ± s) shown are the average of at least 6 independent measurements. The data were performed using one-way analysis of variance (ANOVA), followed by Student two-tailed t test. Significant as compared to model control

*p<0.05

**p<0.01; significant as compared to normal control

^#^p<0.01

^##^p<0.01.

### cFGF-21 Protects Liver against STZ-Induced Damage in Diabetic Mice

**Fig 7:**
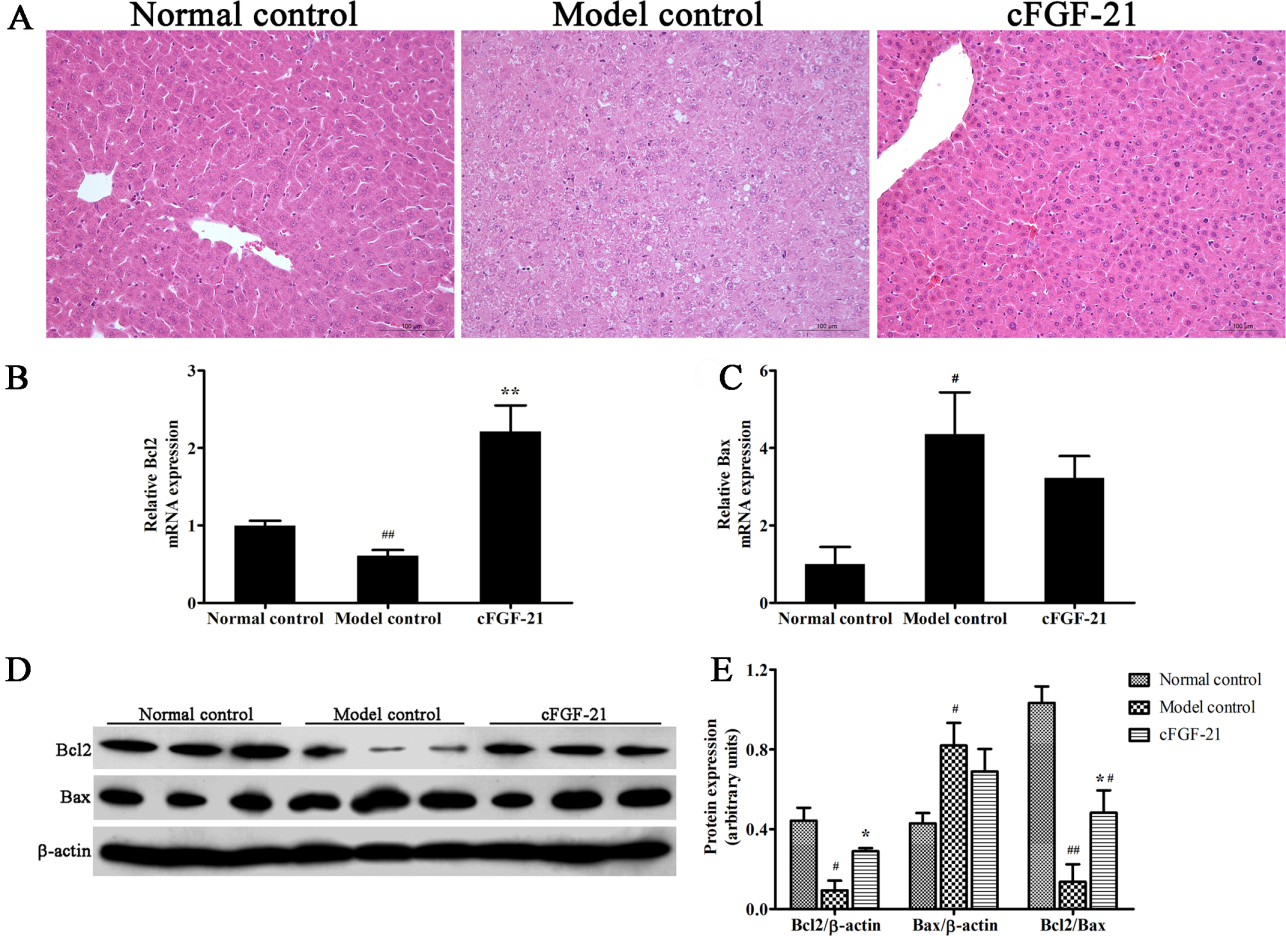
cFGF-21 protects liver against STZ-induced damage. (A) H&E staining of livers. (B) Realtime PCR analysis of the expression of Bcl2. (C) Realtime PCR analysis of the expression of Bax. (C) Western blot analysis of the the expressions of Bcl2 and Bax. (D) The relative the expressions of Bcl2 and Bax levels. The relative the expressions of Bcl2 and Bax levels were expressed as the ratio Bcl2/beta-actin and Bax/beta-actin. The bands were derived from three blots and analyzed with Image pro plus. The data were performed using one-way analysis of variance (ANOVA), followed by Student two-tailed t test. Data represent the mean±SD. significant as compared to model control, *p<0.05, **p<0.01; significant as compared to normal control, ^#^p<0.01, ^##^p<0.01.

H&E staining of livers showed that cFGF-21 protects liver against STZ or hyperglycemia induced damage. H&E staining didn’t reveal any liver damage in normal control and cFGF-21 group. However, there was clear liver vacuolar degeneration in the cytoplasm, hepatic venous passive congestion and pyknosis in the nucleus in the model group (Fig 7A). qPCR and western blotting were used to analyze the expression of apoptosis-related genes (Bcl2 and Bax). Results showed that the expression of Bcl2 decreased significantly in model control compared with normal control, whereas the expression of Bcl2 increased significantly in cFGF-21 group compared with model control. Both expression of Bax in model control and cFGF-21 group increased significantly compared with normal control, and there was no significant difference of Bax expression between model control and cFGF-21 group. However, the Bcl2/Bax ratio in cFGF-21 group increased significantly compared with model control, of which Bcl2/Bax ratio was lower than the Bcl2/Bax ratio in normal control (Fig 7B–7E). Additionally, the ALP, ALT, ASP and GGT levels in model control increased significantly, whereas ALP, ALT and ASP levels were attenuated significantly after treatment with cFGF-21 (Table 3).

**Table 3:** Impact of cFGF-21 on serum ALT, AST, ALP and GGT in mice.

	ALT(IU/L)	ALP(IU/L)	AST(IU/L)	GGT(IU/L)
Normal control	71.17±5.77	54.33±2.76	125.33±9.85	1.17±0.17
Model control	157.33±17.71^##^	166.00±20.66^##^	283.83±42.26^#^	3.67±0.95^#^
cFGF-21	90.67±7.34*	68.17±6.40**	211.33±27.70	1.83±0.31*

The values (x ± s) shown are the average of at least 6 independent measurements. The data were performed using one-way analysis of variance (ANOVA), followed by Student two-tailed t test. Significant as compared to model control

*p<0.05

**p<0.01; significant as compared to normal control

^#^p<0.01

^##^p<0.01.

### cFGF-21 Protects Liver against STZ or Hyperglycemic Induced Damage Associated with Inhibiting Oxidative Stress in Diabetic Mice

**Fig 8:**
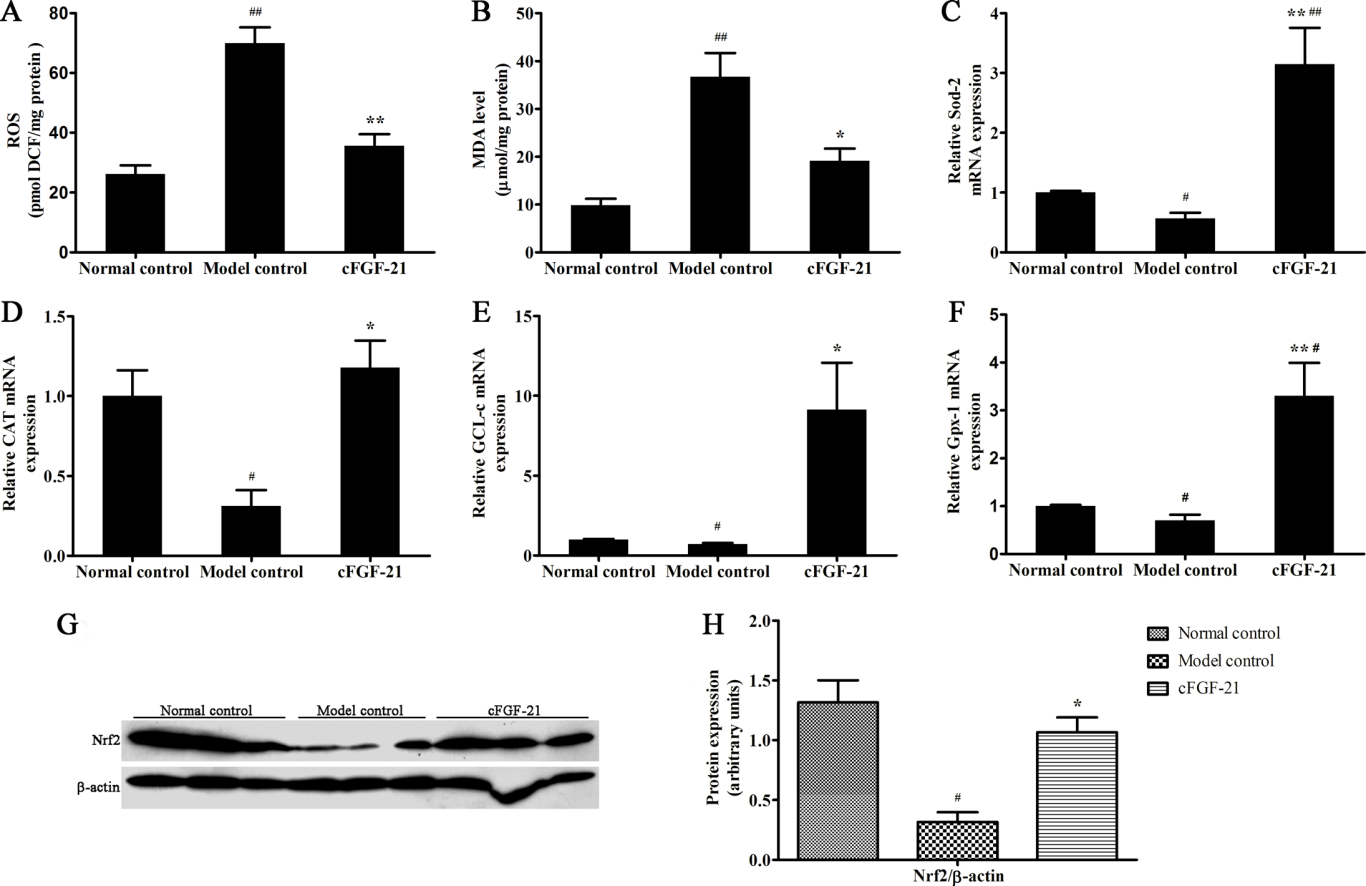
cFGF-21 inhibits STZ-induced oxidative stress in the livers. (A) The ROS content in the livers. (B) The MDA content in the livers. (C-F) The expressions of the antioxidative enzymes (Sod-2, CAT, GCL-c and Gpx-1). (G) Western blot analysis of the expression of Nrf2. (H) The relative the expression of Nrf2 level. The relative the expression of Nrf2 level was expressed as the ratio Nrf2/beta-actin. The bands were derived from three blots and analyzed with Image pro plus. The data were performed using one-way analysis of variance (ANOVA), followed by Student two-tailed t test. Data represent the mean±SD. significant as compared to model control, *p<0.05, **p<0.01; significant as compared to normal control, ^#^p<0.01, ^##^p<0.01.

The ROS and MDA contents in model control increased significantly compared with normal control, and the ROS and MDA contents in cFGF-21 group decreased significantly compared with model control (Fig 8A and 8B). Realtime PCR results showed that the expressions of the antioxidative enzymes (Sod2, CAT, GCL-c and Gpx-1) of the livers decreased significantly in model control compared with normal control, and the expressions of the antioxidative enzymes (Sod2, CAT, GCL-c and Gpx-1) of the livers were up-regulated significantly in cFGF-21 group compared with model control (Fig 8C–8F). Western blotting showed that the expression of Nrf2 in model control decreased significantly compared with normal control. However, when treated with cFGF-21, the Nrf2 expression increased significantly compared with model control (Fig 8G and 8H). Besides, the activities of the antioxidative enzymes in model control decreased significantly compared with normal control, whereas the activities of the antioxidative enzymes increased significantly in cFGF-21 group compared with model control (Table 4).

**Table 4:** Impact of cFGF-21 on hepatic activities of antioxidative enzymes in mice.

	SOD	CAT	GR	GSH-Px(U/mg protein)
Normal control	92.85±5.75	76.23±5.27	359.02±14.91	758.65±32.11
Model control	37.52±4.90^##^	26.03±2.44^##^	148.35±9.55^##^	198.18±15.37^##^
cFGF-21	106.33±6.76**	58.02±4.07**^#^	274.35±12.87**^##^	458.40±20.80**^##^

The values (x ± s) shown are the average of at least 6 independent measurements. The data were performed using one-way analysis of variance (ANOVA), followed by Student two-tailed t test. Significant as compared to model control

**p<0.01; significant as compared to normal control

^#^p<0.01

^##^p<0.01.

### cFGF-21 Exhibits Hypoglycemic Effect in Diabetic Dogs

**Fig 9:**
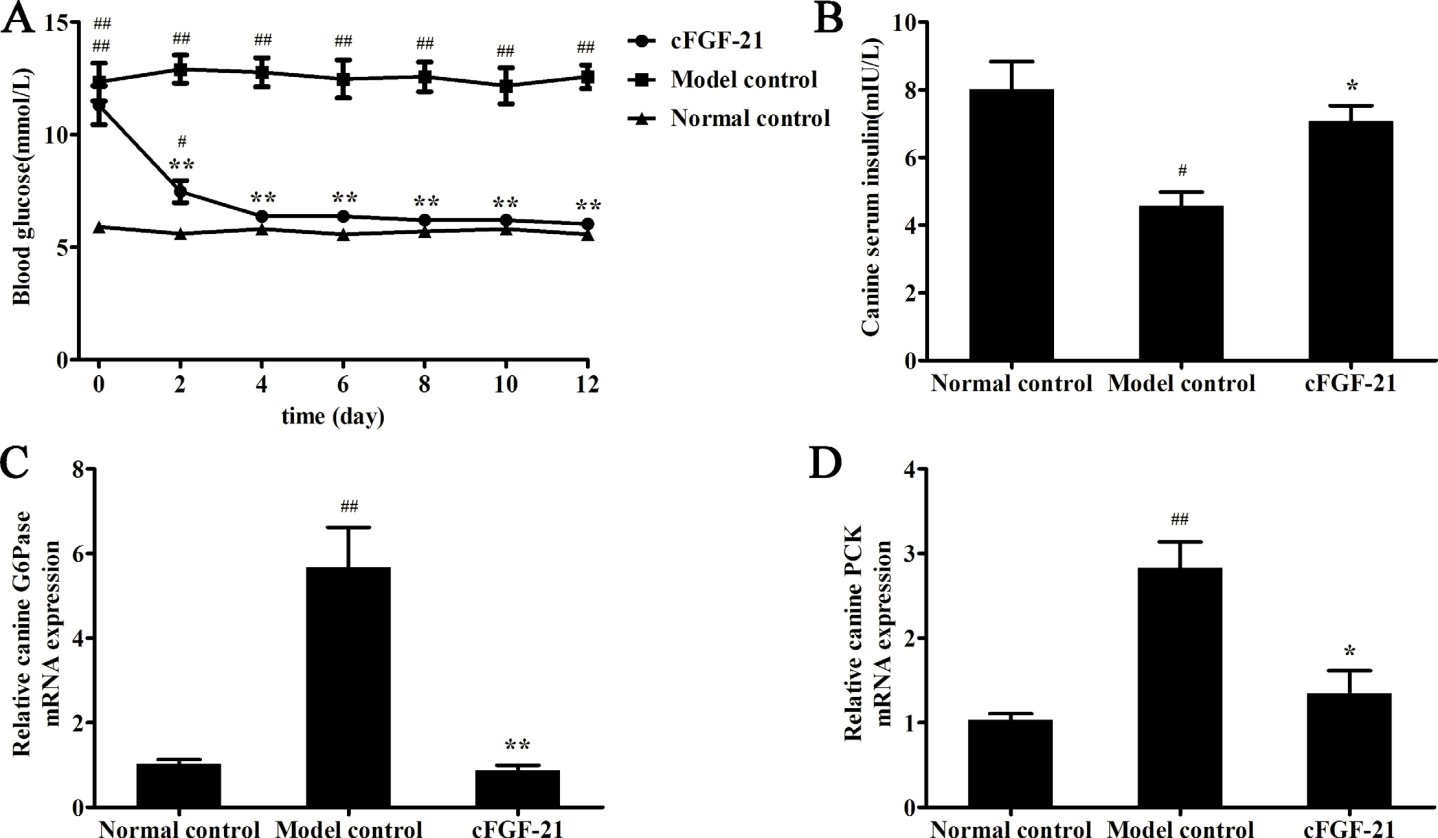
cFGF-21 exhibits hypoglycemic effect in diabetic dogs. (A) The FBG levels of the dogs in different groups during this experimental period. FBG levels of the dogs in different groups every two days. (B) The serum inslin level of the dog in different groups. The dogs sacrificed at the end of the experiment, and the blood was collected for serum insulin measurement. (C) Realtime PCR analysis of the expression of G6Pase. (D) Realtime PCR analysis of the expression of PCK. The data were performed using one-way analysis of variance (ANOVA), followed by Student two-tailed t test. Data represent the mean±SD. significant as compared to model control, ^*^p<0.05, ^**^p<0.01; significant as compared to normal control, ^#^p<0.01, ^##^p<0.01.

Diabetic dogs were induced by STZ at a dose of 25 mg/kg (10 mmol/L Na-citrate buffer, pH 4.0), and the fasting blood glucose (FBG) of the dogs continuously exceeded 11.1 mmol/L the serum insulin level of the dog decreased significantly compared with healthy dogs were thought as diabetic dogs (data not shown). Results showed that treatment with cFGF-21 reduced the blood glucose to near the normal level compared with model control (Fig 9A). Measurement of serum insulin level showed that the serum insulin level of model control decreased significantly compared with normal control; treatment with cFGF-21 significantly improved the serum insulin level of the dogs in cFGF-21 group compared with model control (Fig 9B). qPCR analysis showed that cFGF-21 down-regulated the expressions of G6Pase and PCK significantly compared with model control, of which expressions of G6Pase and PCK increased significantly compared with normal control (Fig 9C and 9D).

## Discussion

Developing alternative therapeutic drugs for canine DM is attractive for many reasons, such as the morbidity of DM in dogs is increasing [4]; the reluctance to perform injections lead to a lot of dogs being euthanized rather than being treated [17,18]. If the owners of dogs decide to treat their diabetic dogs, they will experience a heavy treatment burden of injection [19,20]. Therefore, insulin is not the best choice for treating canine diabetes. Drugs with a long-acting hypoglycemic effect on the DM is needed for the diabetic dogs.

The present study developed a recombinant plasmid pSUMO-cFGF21 that could be used for prokaryotic expression of recombinant cFGF-21 protein. The prokaryotic expression vector—pSUMO vector was seclected to produce recombinant proteins, as previous study has demonstrated that high-level expression of soluble recombinant FGF21 protein can be obtained using pSUMO vector [21]. After expression and purification, the soluble cFGF-21 protein with high purity was obtained. Due to the cFGF-21 sequence exhibited approximately 80% homologous identity to the mouse FGF-21 sequence and the functional regions between the two sequences were almost same, we speculated that cFGF-21 could exhibit its biological activity in mouse cells or mouse. cFGF-21 exhibited good biological activity on improving glucose uptake in 3T3-L1 cells. The *in vivo* activity of cFGF-21 was detected in diabetic mice/dogs. Results in mice showed that treatment with cFGF-21 reduced the blood glucose level to near normal level, and the HbA1c level of the diabetic mice decreased significantly after treated with cFGF-21 compared to diabetic mice without treatment. cFGF-21 exhibited long-acting hypoglycemic effect, which was inspiring. Besides, results in dogs showed that treatment with cFGF-21 also reduced the blood glucose level significantly, which was closed to the blood glucose level of healthy dogs. These results suggest that cFGF-21 has the potential become a new therapeutics for diabetic dogs.

Hepatic gluconeogenesis plays an important role in the pathogenesis of diabetes [22]. G6Pase and PCK are the rate-limiting enzymes in gluconeogenesis [23], and the expressions of G6Pase and PCK are used to analyze the gluconeogenesis level in many studies [13,24,25]. The possible reason is that measuring the activities of G6Pase and PCK is not easy. Therefore, we also chose the expressions of G6Pase and PCK to analyze the gluconeogenesis level in this study. To keep energy balance, FGF-21 induces the increase in gluconeogenesis in response to starvation in healthy mice [26]. Besides, some studies have demonstrated that FGF-21 also can inhibit gluconeogenesis in response to the disorder of glucolipid metabolism in diabetic mouse model [13,25]. In this study, we found that the expressions of G6Pase and PCK were up-regulated significantly in the mice/dogs with type 1 diabetes. However, treatment with cFGF-21 reversed this phenomenon. Therefore, inhibiting hepatic gluconeogenesis may be one of the mechanisms of cFGF-21 action. Previously studies reported that STAT3 plays a role in suppressing expression of gluconeogenic genes [27,28]. To investigate whether cFGF-21 mediates gluconeogenesis was associated with STAT3 signaling. We found that treatment with cFGF-21 up-regulated the expression and phosphorylation level of STAT3. This finding supports the notion that cFGF-21 modulates hepatic glucose homeostasis associated with the STAT3 pathway. Previous studies indicates that SOCS3 is one of the transcriptional targets of STAT3, and SOCS3 is regulated by the STAT3 pathway [29,30]. Besides, previous study has demonstrated that STAT3-SOCS3 signaling pathway is associated with hepatic gluconeogenesis [31]. Therefore, in this study, we found that the expression of SOCS3 was up-regulated significantly in diabetic mice after treatment with cFGF-21. This result further proved that STAT3 was activated in diabetic mice when treated with cFGF-21. These results suggest that the mechanism of cFGF-21 ameliorates hyperglycemia associated with inhibiting hepatic gluconeogenesis by regulation of STAT3 signal pathway.

Apoptosis is the major cause of death of insulin-producing beta- cells in type 1 diabetes. In this study, we found that the serum insulin level of the diabetic mice/dogs increased significantly after treatment with cFGF-21. This finding revealed that cFGF-21 improved pancreatic beta-cell function. H&E staining and immunohistochemistry for the pancreases of the mice showed that cFGF-21 improved pancreatic beta-cell survival and secretion of insulin. Additionally, treatment with cFGF-21 inhibited the apoptosis of the pancreases. cFGF-21 attenuated the expressions of apoptosis-related genes (Bcl2 and Bax) of the pancreases and inhibited the STZ-induced oxidative stress level in the pancreases. These results suggest that another mechanism that cFGF-21 ameliorates hyperglycemia associated with improving pancreatic beta-cell survival. Wolf Wente et al have demonstrated that human FGF-21 has no influence on the islet cell proliferation, and protects β-cells from apoptosis via activation of extracellular signal–regulated kinase (ERK)1/2 and Akt signaling pathways in db/db mice [32]. Therefore, cFGF-21 improves pancreatic beta-cell survival maybe associated with extracellular signal–regulated kinase (ERK)1/2 and Akt signaling pathways.

Liver damage is more commonly seen in T1D patients [33,34]. Previous studies have reported that FGF-21 has the ability to protect liver and inhibit oxidative stress [35]. In this tudy, we also found that liver damage in the diabetic mice without any treatment (exhibiting higher levels of ALP, ALT, AST, GGT and more severe pathological damage compared with healthy mice), whereas treatment with cFGF-21 reversed this phenomenon and protected the liver against hyperglycemia induced liver damage. Besides, the livers in the diabetic mice without any treatment stayed state with high oxidative stress level (exhibiting more ROS and MDA contents, lower activities and expressions of antioxidative enzymes and lower expression of Nrf2 comapred with healthy mice). Antioxidative enzymes form the first line of the antioxidant defence to protect the organism from damage induced by oxidative stress [36]. Although oxidative stress normally induces compensatory increase in the activities of antioxidative enzymes, several studies have indicated that lower activities of antioxidative enzymes in streptozotocin-induced diabetic animals [37,38]. Our results further confirm these conclusions. Treatment with cFGF-21 inhibited the oxidative stress with less ROS and MDA contents, exhibited higher activities and higher expressions of antioxidative enzymes and Nrf2 in the livers compared with the mice in model group. These results suggest that cFGF-21 can protect liver against hyperglycemia induced damage and the possible mechanism of its action is associated with inhibiting oxidative stress.

Although cFGF-21 exhibited superiority in treatment of diabetic mice/dogs in this study, one shortcomings that we found during our experiment existed. When the blood glucose of diabetic mice exceeded approximately 25.0 mmol/L, cFGF-21 could not work well. The possible explaination of this phenomenon was that the pancreatic islet of the diabetic mice with blood glucose exceeding 25.0 mmol/L destroyed completely, and cFGF-21 could not“repair”them.

In conclusion, this study provides a new approach to treating canine diabetes and the cFGF-21 represents a promising candidate for this canine diabetes therapeutics. The mechanism that cFGF-21 ameliorates hyperglycemia associated with inhibiting hepatic gluconeogenesis by regulation of STAT3 signal pathway and improving pancreatic beta-cell survival.

## Supporting Information

S1 Dataset The data of glucose uptake, hypoglycemic effect of cFGF21, OGTT, serum INS and HbA1c in 3T3-L1 cells and mosue experiment. (XLS)

S2 Dataset The data of mRNA expressions of G6Pae, PCK, STAT3, SOCS3, Bcl2, Bax, CAT, GCL-c and Gpx-1 in mouse experiment. (XLS)

S3 Dataset The data of ROS and MDA contents in mouse experiment. (XLS)

S4 Dataset The data of antixoidative enzymes in mouse experiment. (XLS)

S5 Dataset The data of liver function in mouse experiment. (XLS)

S6 Dataset The data of the relative expressions of the SOCS3, G6Pase, PCK, Bcl2, Bax, Nrf2 and the relative phosphorylation level of STAT3 in mouse experiment. (XLS)

S7 Dataset The data of the hypoglycemic effect of cFGF21, the serum INS, HbA1c, the expressions of G6Pase and PCK in dog experiment. (XLS)
